# Cancer histotypes and trends in Azare, Northeast Nigeria: impact of diagnostic support disparity in data reporting

**DOI:** 10.3332/ecancer.2023.1538

**Published:** 2023-04-24

**Authors:** Uchenna Simon Ezenkwa, Mohammed Ibrahim Imam, Maimuna Orahachi Yusuf, Abdullahi Sani Giade, Iragbogie Al-Mustapha Imoudu, Dauda Abubakar Katagum, Bala Muhammad Audu

**Affiliations:** 1 Department of Histopathology, Federal Medical Centre Azare, Bauchi State Nigeria 751101; 2 Department of Pathology, Bayern University/Aminu Kano University Teaching Hospital, Kano, Nigeria 700233; 3 Department of Pediatrics, Federal Medical Centre Azare, Bauchi State Nigeria 751101; 4 Department of Surgery, Federal Medical Centre Azare, Bauchi State Nigeria 751101; 5 Department of Obstetrics and Gynaecology, College of Medicine, Federal University of Health Sciences, Azare Bauchi State Nigeria 751101; a https://orcid.org/0000-0002-7022-8268

**Keywords:** cancer histotypes, trends, diagnostic disparity, cancer data reporting, Northeast Nigeria

## Abstract

**Background:**

Definitive, affordable, and timely diagnosis of cancer is key to providing data for surveillance and control programmes. Care disparities have been shown to contribute to poorer survival, especially in resource-constrained populations. Here, we describe the profile of histologically diagnosed cancers in our hospital and highlight the possible effects of inadequate diagnostic support on data reporting.

**Methods:**

We designed a retrospective cross-sectional descriptive study to review histopathology reports archived at the Department of Pathology of our hospital spanning from January 2011 to December 2022. Cases diagnosed as cancer were retrieved and classified by systems, organs and histology types alongside the patient’s age and gender. The trend in the volume of pathology requests and the corresponding malignant diagnosis yield over the period was also documented. Data generated were analyzed statistically using appropriate statistics and presented as proportions and means, with the level of statistical significance set at *p* < 0.05.

**Results:**

There were 488 cancers out of 3,237 histopathology requests received within the study period. Of these 316 (64.7%) were females. Overall mean age was 48.8 ± 18.6 years with a peak age at the sixth decade, females being significantly younger (46.1 versus 53.5 years; *p* < 0.001). The top five cancers were breast (22.7%), cervical (12.7%), prostate (11.7%), skin (10.7%) and colorectal cancers (8%). Among females, breast, cervical and ovarian cancers predominated, whereas prostate, skin and colorectal cancers, were commonest among males in decreasing order. Paediatric malignancies accounted for 3.7% of all the cases, most being small round blue cell tumours. The volume of pathology requests rose remarkably from 95 cases in 2014 to 625 cases in 2022 with a corresponding increase in cancer case diagnoses.

**Conclusion:**

Cancer subtypes and ranking in this study are similar to those from urban populations in Nigeria and Africa, despite the low number of cases recorded. Efforts to reduce the disease burden are warranted.

## Introduction

Cancer is a disease generating attention globally owing to the need to stem its rising incidence and improve survival. The GLOBOCAN data estimate that 19.3 million incident cases with 10.0 million deaths were recorded globally in 2020, representing a 7% increase in incidence and a 3% increase in mortality over that recorded in 2018 [1, 2]. Risk factors associated with the development of cancer are largely lifestyle-dependent and vary according to sociodemographic factors [3]. Likewise, the disparity in diagnosis and treatment contributes to poorer survival in low-middle-income countries [4, 5]. Africa accounted for about 6% of the world’s incident cases in 2020 which were derived from cancer registries [6]. Both population and hospital-based data from Nigeria have reported a rising number of cancer cases in Nigeria [7, 8–10]. There is however a dearth of comprehensive data describing the spectrum of malignancies in our immediate environment in the Northeast Nigeria except for a previous report of gynaecological cancers [11]. As our objective, we set to describe cancer histological types, the trends in diagnosis and the influence diagnostic support availability could have on data reporting. Understanding our situation will provide invaluable data for awareness propagation and risk-modifying strategies in addition to contributing to the growing knowledge base on cancer among Nigerians living in our locale.

## Materials and methods

This retrospective cross-sectional descriptive study was conducted at the Federal Medical Centre Azare, Bauchi State Northeast Nigeria. Azare is situated at latitude 11^°^40′27ʺ North and longitude 10^°^11′28ʺ East with an estimated population of about 110,452 using the 2007 census figures, the inhabitants being mostly agricultural in occupation consisting of pastoral and crop farming [12]. The hospital is a 350-bed capacity tertiary health institution and serves as a referral centre for all the primary and secondary health care centres in the communities and towns from neighbouring states, while the two closest tertiary hospitals with histopathological services were located about 200 km away each. Histopathology reports archived at the Department of Pathology of our hospital from January 2011 to December 2022 were reviewed.

The majority of requests (about 75%) were derived from primary hospital patients who accessed healthcare directly while fewer patients were referred from primary and secondary health centres for further management or investigation. The average cost of histopathology testing over this period was N1,500 (N1,200 to N2,800), and available oncology services were mainly surgical excision with curative intent and chemotherapy. For this study, samples reported as cancers were retrieved with the documented patient age, gender and cancer site, and classified into systems, organs and histology types. The trend in annual case requests and cancer diagnosis was additionally determined.

The Statistical Package for Social Sciences version 20 was used to analyse the data generated. Descriptive statistics was applied to the data to determine the proportions of variables. The difference in means between continuous variables was also analysed using the student *t*-test function. The results were presented in prose, figures and tables.

All patient data were fully deidentified prior to analysis. This study adhered to the Helsinki Declaration on Research on human subjects of 1964 and its later amendments. Institutional review board approval was received from the hospital’s ethics committee prior to the commencement of the study.

## Results

**Figure 1.**
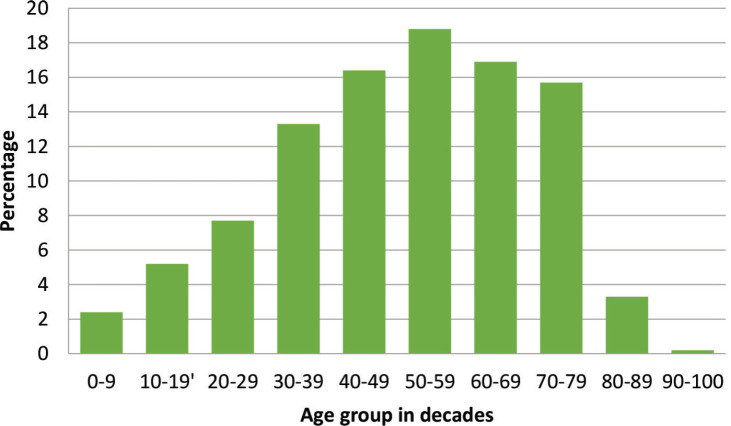
Age group of cancer patients in decades with a progressive increase in cancer occurrence as age advances.

There were 3,237 histopathological requests within the time period. A total of 488 cancers were diagnosed histologically during this period representing 15.1% of all received histological specimens. Of these malignant cases, there were 316 females and 172 males giving a female-to-male ratio of 1.8:1. Overall mean age was 48.8 ± 18.6 years, females being significantly younger (46.1 versus 53.5 years; *p* < 0.001). Age group by decades of the patients is presented in Figure 1 with the peak age being in the sixth decade.

Table 1 shows the list of all cancers documented. Overall, the top five cancers were breast (22.7%), cervical (12.7%), prostate (11.7%), skin (10.7%) and colorectal cancers (8.0%). There were cases of metastatic cancers whose primary sites were not resolved. These were less than 1% of the cases. Among females, breast, cervix and ovarian cancers predominated, while prostate, skin, and colorectal cancers, were commonest among males in descending order (Tables 2 and 3). Gallbladder, soft tissue, skin and sinonasal cancers were about equal in both genders. Paediatric malignancies accounted for 3.7% of the cases, most being small round blue cell tumours.

**Figure 2.**
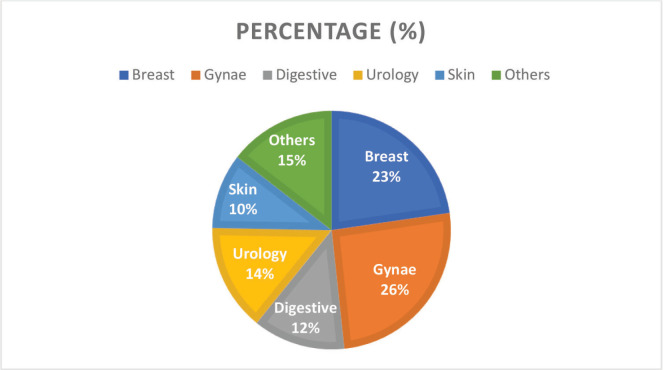
Cancer types by the system and their frequencies.

**Figure 3.**
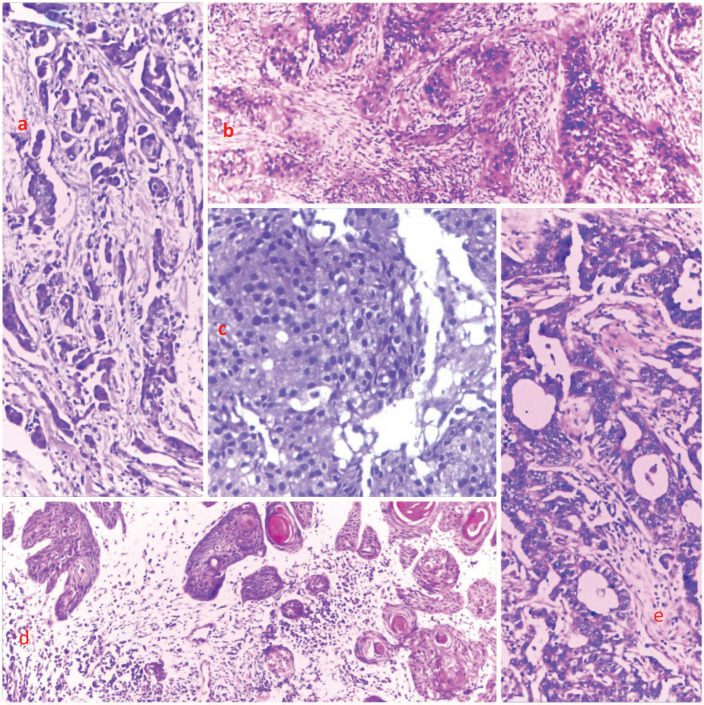
Photomicrographs showing the histological features of selected cases from the top five cancers. (a): Breast cancer, (b): cervical cancer, (c): prostate cancer, (d): skin cancer and (e): and colon cancer, respectively. H&E, (a): ×40, (b): ×100, (c): ×400, (d): ×40 and (e): ×100.

Grouped according to systems, gynaecological cancers were the commonest, accounting for about 26% of all the cases (Figure 2). Each system group had different histological types of cancers. Figure 3 shows a photomicrographic representation of the top five cancers in both genders combined as seen in this study.

### Cancer by system

The malignancies seen here were further classified according to the organ or system involved and the most common five groups are presented in more detail below. These groups include the gynaecological, breast (mammary), digestive, urological and skin (dermatological) cancers. In addition, paediatric cancers are also described to highlight the spectrum of malignant tumours with similar phenotypes but occurring in different organs and systems of the body.

### Gynaecological cancers

This was the commonest system involved in cancer accounting for 125 (25.6%) of all cases. They were comprised of ovarian, uterine, cervical and vulvovaginal cancers. The median age of these patients was 50 years ranging from 17 to 75 years. Over half of the cases occurred in the fifth to seventh decades of life.

Cervical cancer was the most predominant in this category accounting for 62 (49.6%) cases. The majority of these were squamous cell carcinoma seen in 51 (85%) of persons, while adenosquamous and adenocarcinomas account for three cases each. Two cases of clear cell carcinoma and one case of sarcoma were also documented.

Thirty-four (28.1%) of gynaecological cancers were from the ovary. The majority (31 out of 35) of these were cystadenocarcinomas out of which, 7 were mucinous varieties. There were also two cases of dysgerminoma and one each of yolk sac tumour and malignant Brenner’s tumour.

Uterine endometrial and myometrial cancers were 23 in number out of which were 14 endometrioid carcinomas, 4 choriocarcinomas, 3 squamous cell carcinomas, 2 leiomyosarcomas and 1 endometrial stromal sarcoma.

There were four cases of vulvovaginal cancers, two of which were choriocarcinomas, and one each of rhabdomyosarcoma and squamous cell carcinoma.

### Breast cancer

Breast cancer was the commonest type of cancer from a single body organ (111 cases). About 96.4% occurred in women; 4 (3.6%) cases were seen in men. The median age was 45 years (18–80 years) with a peak in the fifth decade. About 97.3% of the cases were histologically invasive carcinoma of no special type. Other variants observed were secretory carcinoma, sebaceous carcinoma and non-Hodgkin lymphoma and these accounted for 0.9% of all breast cancers each. Laterality was about equal between the right and left breast both having 40.2% and 41.2% each. One patient had bilateral breast cancers while laterality was not documented in about 18 cases.

### Urological cancers

Cancers of the kidneys, urinary bladder and prostate are presented in Table 1. The renal malignancies were seen more in females (four out of six), with five cases being renal cell carcinoma and one documented as nephroblastoma histologically. Six of the eight cancers of the urinary bladder were seen in men; these cancers were classified as two squamous cell carcinomas and six urothelial carcinomas. All 57 cases of prostate cancers were adenocarcinomas. Overall, there were 71 urological cancers recorded.

### Digestive system cancers

Sixty-one cases were cancers involving the digestive system comprising malignancies of the oral cavity, salivary glands, stomach, biliary, colon and anorectum. These were derived from patients aged 6–90 years with a median of 50 years. The overall peak age was in the age group 50–59 years and most were seen in men with a ratio of 1.6:1.

There were nine cancers of the oral cavity; five of these were in the salivary glands (four parotid and one minor salivary glands), classified histologically as mucoepidermoid carcinomas in three cases and adenoid cystic carcinomas in two cases. The remaining four oral cancers were poorly differentiated in three variants while one was a high-grade sarcoma, and these were distributed two cases each in the palate and the oropharynx.

The gastric cancers seen were all adenocarcinomas and occurred in six males and three females. Three of these patients were in the fourth decade of life, two in the seventh while four patients had age distribution between these two.

Two gallbladder adenocarcinomas were recorded, one in each gender and both patients being in their early sixties. There was one case each of jejunal and appendix cancer, 25 colonic and 14 rectal cancers. Their histology varied, two being neuroendocrine carcinomas, one non-Hodgkin lymphoma while the others were adenocarcinomas. There were more males with a little over a quarter of the patients being less than 50 years of age at the time of diagnosis.

### Skin cancers

There were 50 cases of skin cancer with slight female preponderance (female/male ratio 1.1:1) and a median age of 51 years (range 13–80 years). Cutaneous melanoma ranked second with 12 (25%) cases after squamous cell carcinoma which had 29 (56%) cases among all the observed cancers. Three cases of dermatofibrosarcoma protuberance and one of basal cell carcinoma in addition to five undifferentiated carcinomas were also documented. About 60% of these tumours occurred on the lower limb (thigh, leg, foot).

### Paediatric cancers

Paediatric cancers were seen in different organs listed in Tables 1–3. As presented in Table 4 below, there were 19 cases derived from 12 males and 7 females with a median age of 8.5 years (range 4–7 years). Cancer with the highest proportion was lymphoma; others were rhabdomyosarcoma, nephroblastoma, retinoblastoma and squamous cell carcinoma on the hand. Additionally, there were two cases described as small round blue cell tumours and one case of skin nodules with epithelial features but not further characterised.

### Cancer volume by year and trend over the study period

**Figure 4.**
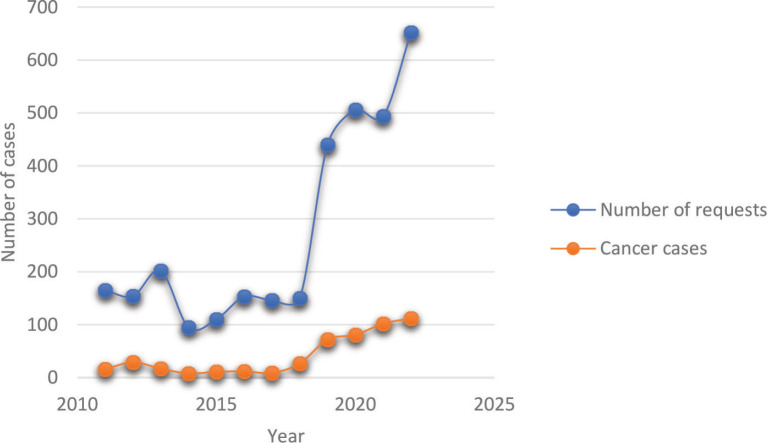
Trends of histopathological requests and cancer diagnosis over the reviewed period. The least annual volume was in 2014 while a steady increase in both requests and cases was seen from 2019 onwards.

The overall histopathology request volume seen each year and the trend over the years are shown in Figure 4. Observably, the early part of the curve had fewer number of requests averaging around 200 documented cases with a steady rise in the later years. Expectedly, the number of diagnosed cancer cases reflected the volume of requests proportionately.

## Discussion

The cancer burden is increasingly reported in developing communities transitioning to more Westernised lifestyles [2]. Our study has rather shown low numbers of cancer cases from all body sites seen over a decade in our population. In Africa, Nigeria ranks second after Egypt with the highest number of cancer cases and deaths [6]. There are few population-based cancer registries in Africa, hence, actual cancer incidence and mortality in the population may be under-represented [13]. It is projected that by the end of the present decade, cancer mortality could rise to about a million in the continent [14]. Most of these deaths may be unaccounted for as a result of inadequate data coverage of remote underserved communities such as ours. The present study not only adds to the existing data from our country but further highlights the particular challenges being faced in underserved localities in establishing the true burden of this disease.

Although hospital and microscopy based, the tumour histotypes and their ranking documented here compare favourably with results derived from cancer registries from Nigeria [15]. Indeed, there is a similarity between our top five cancers and those seen in the population-based cancer registries in Nigeria, Africa and globally [2, 6, 7]. The index population is less urban compared to those with cancer registries cited previously and the occupation is mainly agriculture-based, suggesting a less sedentary lifestyle and diets likely high in whole grains and fibre. We think that the effect of these well-known risk factors of carcinogenesis, in addition to obesity and smoking, may not completely explain the similar cancer profiles between our community and those in more developed societies [3]. There is, therefore, a need to investigate probable environmental, cultural or genetic factors driving these tumours in our locality. Also, the absence of lung cancer in the top five seen in Africa and Nigeria may indicate its lower prevalence in the African population or underdiagnosis of these cases [6, 7, 16]. The role of unsafe sexual practices on the high proportion of cervical cancer seen may need validation [3].

The prevailing higher number of breast and gynaecological cancers explains the female preponderance in the report. This is in concordance with Africa continent data but contrary to the global outlook where more males had higher incidence rates in 2020 [2, 6]. Mohammed *et al* [17], in a report from Kano, a neighbouring state to this study site, showed a slight male preponderance while that by Sahabi and Abdullahi [18], from Sokoto Nigeria had more females. These studies, also based solely on histologically diagnosed cases, likewise had more gynaecological and breast cancers [17, 18]. These variations signify that besides gender-based tumours, most other malignancies were more prevalent among males as was also found in the index study.

The overall mean age of the patients in the present study was less than 50 years suggesting a preponderance among the younger population, similar to a study by Uchendu [8] from the southern part of the country; in addition, we found a significantly younger female population due to higher proportions of female gender-related malignancies. Perhaps this could be ascribed to the prevalent young age of the population. Cancer in the young is indeed assuming alarming proportions the world-over [19]. The increase in observed cases as age increased is in tandem with the established knowledge that cancer incidence increases with age and this is true in every population [20]. However, depending on a population’s life expectancy, more cases may appear prevalent at an either older or younger age. It may therefore be expected that with an increase in life expectancy, the age of our demographics may tilt towards the older age group. In this regard, as more individuals survive infectious and cardiovascular diseases, there will be more people living in old age, and this, combined with the adoption of cancer-promoting lifestyles, may cause more cases of cancer to emerge [20, 21].

The low number of documented cancer cases in our report could be a reflection of the difficulties encountered in navigating the diagnostic pathway, while the trend in the volume of histopathological requests represents periods when the centre had minimal surgical pathology support in the earlier years transitioning to later years with established substantial histopathological workflow, and not due to increase in disease incidence. This shows that having a functioning pathology laboratory is vital in generating good quality data for intervention policy formulation because where this is lacking, the true burden of the disease will be obscured and this is evident in poorer underserved communities in Africa [13, 22]. And when these facilities are located far from the patients, as was the case in our experience, patronage becomes difficult, especially when the cost is borne by the patients [23]. Other authors from Nigeria, even within cities, have noted a negative impact of point-of-care distance on diagnostic service uptake [17]. In effect, these factors further worsen the disparity existing between rural and urban dwellers in cancer care with associated poorer outcomes among the rural population [4, 24].

Certain cancer types such as liver, lung and brain cancers were not represented in this study, perhaps due to the inability to biopsy or resect these tumours. We also observed tumours with unknown primary anatomical sites that were not further investigated owing to a lack of ancillary testing support and this has implications for determining what treatment the patient should receive. Besides its effect on care, tissues and data for genomic research into these malignancies from our environment to understand their characteristics will be lacking. Therefore, building capacity for adequate patient care in such settings should include training skilled oncology personnel for all levels of care in addition to creating the setting for cutting-edge research to prevent, detect or treat these cancers [25–28]. Also, immunohistochemistry and molecular testing of tumours should begin to be prioritised in poorer communities in order to reasonably identify each tumour type and determine predictive and prognostic features for individual patients [29, 30].

Despite the limitations occasioned by the histopathological basis of our data, the percentage of malignant diseases among our received samples (15.1%) did not remarkably differ from others reported in the country with proportions ranging from 13.7% to 24.4% in various local studies [8, 31–33]. The similarities in patients’ demographics, tumour histotypes and profiles between these studies and ours may suggest a similar cancer burden too, even though these other studies had higher volumes of surgical specimens and were derived from more urban cities. A second limitation is the absence of data on tumour stage at diagnosis that was not overcome due to the retrospective nature of the study. However, the presence of few cases of suspected metastatic diseases, without known primary site of origin, shows that there were cases with very advanced disease stages, further suggesting a need to diagnose oncology diseases early and appropriately stage them for therapeutic and prognostic purposes. Enhancing screening services at the primary healthcare level to detect premalignant lesions and early-stage cancers will help in changing this situation. Also, establishing a cancer registry within the hospital with a mandate to follow up with patients even at the community level will help make survival data available for proper prognostic interrogation of this disease.

## Conclusion

The cancer disease burden in our population appears low, however, it shares similar histological profiles when compared to other indigenous and non-indigenous data which may suggest under-reporting in our environment given that laboratory diagnostic support is inadequate. This might be masking a silent epidemic that could overwhelm an already weak health system if this trend is not reversed. Dedicated efforts are therefore required to set up responsive oncology services that will enable underserved communities access to quality cancer care and provide data for policymaking, novel adaptable intervention programmes and translational research to eliminate some, if not all the cancer types from the population.

## Conflicts of interest

The authors declare that they have no conflict of interest to declare with regard to this study.

## Source of funding

None.

## Institutional review

This study was reviewed and approved by the Federal Medical Centre Azare Ethics, Research and Review Committee before the commencement of the study.

## Author contribution

USE, BMA conceived the study. USE, MII retrieved and reviewed the histopathological classification of the cases. USE, MII, IAI, MOY, ASG reviewed the data and statistical analysis. USE, MOY, IAI produced the first manuscript. DAK, BMA reviewed the manuscript extensively. All the authors read and approved the final submission.

## Figures and Tables

**Table 1.** Cancer types by organs in decreasing frequency and percentage for both gender and all ages.

Organ	Frequency (f)	Percentage (%)
Breast	111	22.7
Cervix	62	12.7
Prostate	57	11.7
Skin	50	10.2
Colorectal	39	8.0
Ovary	35	7.2
Uterus	24	4.9
Soft tissue (sarcomas)	16	3.3
Lymphoma	17	3.5
Ocular	14	2.9
Unknown primary	9	1.8
Oral	9	1.8
Stomach	9	1.8
Bone	7	1.4
Bladder mass	8	1.6
Kidney	6	1.2
Sinonasal	5	1.0
Vulvovaginal	4	0.8
Gallbladder	2	0.4
Thyroid	2	0.4
Appendix	1	0.2
Small intestine	1	0.2
Total	488	100

**Table 2.** Female cancers by organ, frequency and percentage.

Organ	Frequency (f)	Percentage (%)
Breast	107	33.8
Cervix	62	19.6
Ovary	35	11.3
Skin	25	7.9
Uterus	24	7.6
Colorectum	15	3.7
Soft tissue sarcomas	7	2.2
Lymphoma	6	1.9
Ocular	6	1.9
Unknown primary	6	1.9
Kidney	4	1.3
Vulvovaginal	4	1.2
Bone	3	09
Oral	3	09
Stomach	3	0.9
Thyroid	2	0.6
Sinonasal	2	0.6
Bladder	2	0.6
Gallbladder	1	0.3
Total	316	100

**Table 3.** Male cancers by organ, their frequency and percentage of the occurrence.

Organ	Frequency (f)	Percentage (%)
Prostate	57	33.1
Skin	25	14.5
Colorectum	24	14.0
Ocular	8	4.7
Soft tissue	9	5.2
Unknown primary	3	1.7
Lymphoma	11	6.4
Stomach	6	3.5
Oral	6	3.5
Bladder	6	3.5
Bone	5	2.9
Sinonasal	3	1.7
Breast	4	2.3
Kidney	2	1.2
Gallbladder	1	0.6
Appendix	1	0.6
Jejunum	1	0.6
Total	172	100

**Table 4.** Paediatric cancer by histologic type.

Cancer type	Frequency (f)	Percentage (%)
Lymphoma	7	36.8
Rhabdomyosarcoma	5	26.3
Nephroblastoma	2	10.5
Retinoblastoma	1	5.3
Small round blue cell tumour NOS	2	10.5
Squamous cell carcinoma	1	5.3
Carcinoma of unknown primary	1	5.3
Total	19	100

NOS – Not otherwise specified
